# LWRR: Landscape of Wheat Rust Resistance towards practical breeding design

**DOI:** 10.1007/s44154-025-00232-x

**Published:** 2025-04-14

**Authors:** Jiwen Zhao, Haitao Dong, Jinyu Han, Jingrui Ou, Tiantian Chen, Yuze Wang, Shengjie Liu, Rui Yu, Weijun Zheng, Chunlian Li, Zhensheng Kang, Dejun Han, Qingdong Zeng, Xiaojie Wang, Shengwei Ma, Jianhui Wu

**Affiliations:** 1 State Key Laboratory of Crop Stress Resistance and High-Efficiency Production, College of Agronomy, Northwest A&F University, Yangling, Shaanxi 712100 P. R. China; 2 State Key Laboratory of Crop Stress Resistance and High-Efficiency Production, College of Plant Protection, Northwest A&F University, Yangling, Shaanxi 712100 P. R. China; 3 Yazhouwan National Laboratory, Sanya, Hainan 572024 P. R. China

Dear Editor,

Wheat is one of the most important food crops. In recent years, factors such as global warming have led to the frequent occurrence of extreme weather, altering the epidemic patterns of crop diseases and pests and posing severe challenges to food security (Savary et al. 2019). Wheat yellow rust (YR), also known as stripe rust, is caused by the biotrophic fungus *Puccinia striiformis* Westend f. sp. *tritici* (*Pst*) and remains one of the most devastating diseases threatening global wheat production (Chen 2020; Hovmøller et al. 2023). Genetic resistance breeding has emerged as the most economical and environmentally sustainable approach to the control YR. Over the past decades, researchers have identified numerous disease resistance quantitative trait loci (QTLs) and genes in wheat through bi-parental QTL mapping and genome-wide association studies (GWAS). We compiled 1,125 QTLs/genes from 175 articles and consolidated them into 217 independent QTLs based on genome-wide linkage disequilibrium block characteristics. Additionally, our recent study identified 431 wheat stripe rust resistance QTLs through genome-wide association analysis (Wu et al. 2025). This dataset encompasses 83% (182/217) of previously known independent QTLs. All information about these QTLs is available through online queries in our database. This work provides valuable insights into the genetic architecture of wheat YR resistance and highlights the complexity of its resistance mechanisms.

Despite these advances, several critical challenges hinder the practical application of resistance loci in breeding programs. First, the dispersion of valuable genetic information across hundreds of publications creates significant barriers to accessing and interpreting comprehensive resistance profiles. Second, the complex interactions between resistance loci and their variable effectiveness against different *Pst* races remain poorly understood. Third, identifying candidate genes within QTL regions and analyzing their functions require extensive bioinformatics expertise and computational resources, which are often inaccessible to many breeding programs (Hafeez et al. 2021).

To address these challenges and enhance the effective utilization of YR resistance resources, we have developed Landscape of Wheat Rust Resistance (LWRR, https://wheat.dftianyi.com). Which is a comprehensive web-based platform that integrates extensive phenotypic and genotypic data from 2,191 wheat accessions. This platform is designed to facilitate its application in breeding programs for variety development. LWRR features seven integrated modules, including population structure analysis, trait exploration, GWAS results visualization, QTL information, and candidate gene analysis. For example, entering the sample name "Xinong979" in the search box on the homepage returns details such as its origin, breeding year, phenotype information, and potential stripe rust resistance loci. Similarly, searching for the disease resistance gene *Yr30* provides information on its physical location, associated QTLs, and the frequency of the superior allele across different generations and regions.

To decode wheat YR resistance loci at the level of large-scale population variation, we compiled data from 2,191 publicly available wheat accessions. Through GWAS, we identified 431 QTLs associated with YR resistance (Fig. 1A). By integrating these newly identified loci with previously reported ones (detailed reference information is available on https://wheat.dftianyi.com/?page=References), a meta-analysis revealed a total of 1,125 QTLs for wheat YR resistance. These QTL regions encompass 9,276 candidate genes.

**Fig. 1:**
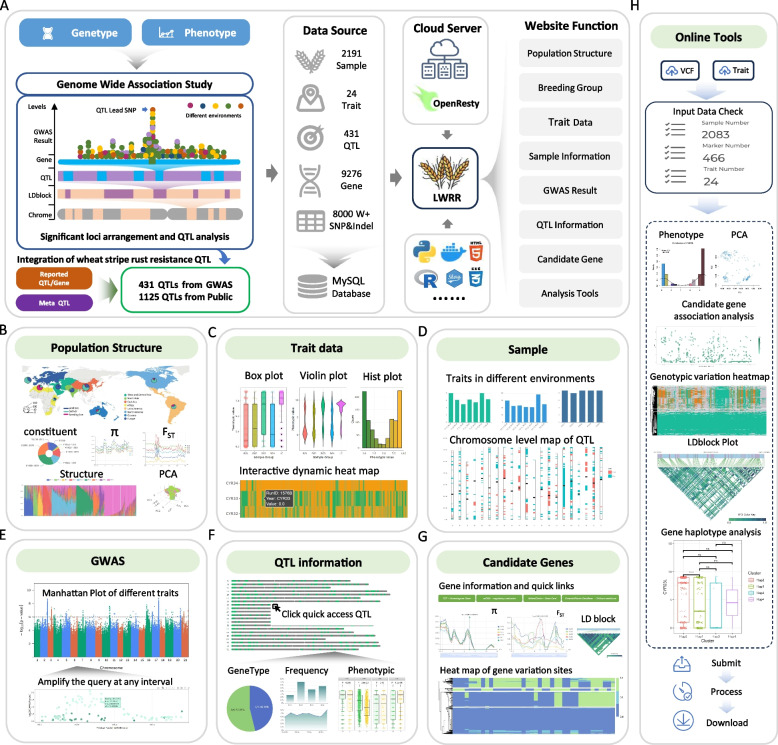
Overview and main functional modules of LWRR. **A** The development process and overall design framework of LWRR, illustrating the workflow from data processing to website construction. **B** The population structure module displays the genetic characteristics of different breeding groups. **C** The trait information module provides an overview of wheat resistance to various stripe rust types, with multiple visualization tools available for user-friendly queries. **D** The sample information module allows users to input a sample Identifier to query origin information, phenotypic values across different environments, and the distribution of resistance loci. **E** The GWAS results module identifies significant loci across phenotypes through large-scale GWAS. Users can explore specific physical intervals in detail. **F** The QTL resistance loci information module enables users to view detailed data on specific QTLs of interest, including inferred changes in their usage frequency. **G** The candidate gene module provides gene information retrieval. A curated collection of 9,276 candidate wheat stripe rust resistance genes is available, including details such as physical location and functional annotation. **H** The cloud-based online analysis tools module allows users to upload customized genotype and phenotype files for candidate gene analysis of single genes, with downloadable analysis results

LWRR features a dynamic, responsive architecture with seven main functional modules: "Population", "Trait", "Sample", "GWAS", "QTL", "Gene" and "Tool", enabling seamless user navigation. With an intuitive online interface, LWRR allows breeders to rapidly access comprehensive information on wheat rust resistance loci, including the distribution of resistance loci in current germplasm and differences in their utilization. Additionally, users can identify the number and identity of rust resistance loci present in specific accessions within the germplasm database.

The "Population" module provides information on wheat accession sources and population structure, enabling comparisons and analyses across different breeding populations (Fig. 1B). Population structure data are presented in three sections: Admixture analysis (for K values ranging from 2 to 9), Principal component analysis, and breeding group (BG) analysis. Users can zoom in to focus on specific samples, and clicking on a sample displays its corresponding information in real time. To facilitate the study of selection and genetic characteristics among different subgroups, we calculated key population genetic statistics, including nucleotide diversity (π), fixation index (*F*_ST_), and Tajima's *D*, at the whole-genome level for different breeding populations and landraces. Additionally, the module features a dynamic query function, allowing users to input any chromosomal region and retrieve detailed genetic data.

The "Trait" module provides YR response data for wheat accessions at both the seedling and adult plant stages (Fig. 1C). Seedling-stage data includes responses to 12 *Pst* races, while adult-plant-stage data covers phenotypic responses under 12 environmental conditions. This module provides basic descriptive statistics for YR response and enables comparisons across different BGs.

The "Sample" module allows users to query an accession's population structure, growth habit (winter/spring), rust disease phenotype, and the presence or absence of rust resistance QTLs using either its Identifier (ID) or name (Fig. 1D). Additionally, the module provides a genome-wide visualization of disease resistance loci for specific accessions, facilitating a comprehensive analysis of resistance loci within their genome. Clicking the target QTL hyperlink automatically directs users to the detailed information interface for further exploration.

The "GWAS" module provides comprehensive GWAS results generated using both the mixed linear model and fixed and random model circulating probability unification methods (Fig. 1E). The results include genome-wide significant variants identified based on a threshold of -log_10_(*P*) > 3. From the Manhattan plot, users can identify or pinpoint significant peak regions and input a chromosome ID and interval to generate a zoomed-in Manhattan plot for the specified region. The module also presents a table listing significant variants within the selected interval, along with their variant effects and *P* values. Additionally, users can directly input a target QTL interval to retrieve the corresponding results.

The "QTL" module provides a comprehensive query interface for all 1,125 QTLs and genes associated with YR resistance (Fig. 1F). Basic information includes QTL location, interval length, and the number of genes within each QTL region. The webpage also displays the lead single nucleotide polymorphism for each QTL, the resistant (R) allele, the R allele frequency across all accessions, and its distribution across eight time periods (pre-1950 to 2020) and four BGs. Additionally, comparisons of YR response between accessions with and without the *R* allele, performed using a t-test, are visualized using box-and-dot plots.

The "Gene" module enables the analysis of candidate genes within YR resistance QTL regions (Fig. 1G). Users can retrieve specific information about wheat disease resistance candidate genes, including their functional annotation and physical location. Additional information on target genes can be accessed from external databases, such as homologous genes from the Triticeae Gene Tribe (Chen et al. 2020), co-expression networks from the wheat Gene Regulatory Network (Chen et al. 2023), and complementary data from WheatOmics (Ma et al. 2021). The module also provides nucleotide diversity and selection signal variations within a 10 Mb region surrounding the gene. In the middle of the page, users can explore variation sites within the gene and examine linkage disequilibrium between different variants. At the bottom, a genotype heatmap of the candidate gene is displayed, with each row representing a different accession to assist in haplotype determination.

The "Tool" module enables candidate gene analysis using user-uploaded genotype and trait files (Fig. 1H). Users can submit data in variant call format-format genotype and phenotype data in Excel format. The LWRR cloud platform performs real-time analyses, including gene variant locus analysis, haplotype analysis, single-marker and haplotype association analyses, and linkage disequilibrium analysis. Once a task is submitted, it enters a computational queue, and a notification appears on the page. During this process, users should avoid closing or refreshing the webpage. Upon completion, the results are displayed in the center of the page. Users should review and download the results promptly, as all temporary data is deleted upon closing the page to ensure data security.

In summary, LWRR is a comprehensive database and analysis platform for wheat YR resistance, built using data from 2,191 global wheat accessions and their phenotypic responses to YR across multiple environments. The platform integrates seven modules, providing key analyses such as population structure, phenotype and genotype exploration, association studies, and customizable analysis tools. LWRR serves as a valuable resource for breeders and researchers, offering both pre-analyzed data and flexible tools to facilitate the exploration of YR resistance in wheat.

## Method

The LWRR panel comprises a diverse and representative collection of wheat accessions from major wheat-growing regions worldwide, including 272 from Africa, 929 from Asia, 640 from Europe, 298 from the Americas, and 52 from Oceania. This panel includes 684 landraces and 1,507 cultivars, representing 70 years of breeding history (1950—2020). Based on distinct YR resistance profiles observed across regions, these cultivars were further classified into four BGs (BG1—BG4). The dataset includes seedling-stage resistance evaluations against 12 *Pst* races (Chinese Yellow Rust physiological race CYR17, CYR23, CYR29, CYR31, Sull-4, Sull-5, CYR32, CYR33, CYR34, V26/GS, V26/SC, and TSA-V5) and adult-plant-stage resistance assessments under 12 environmental conditions. Field trials were conducted in Yangling city (2019—2022) in Shaanxi Province; Jiangyou City (2019—2021) in Sichuan Province; Tianshui City (2019—2021) in Gansu Province; Chongqing City (2021); and Guiyang City (2021) in Guizhou Province.

During the development of LWRR, the R programming language (https://www.R-project.org) was used for data processing and website construction. The frontend was built using Shiny and Bootstrap, with dynamic interactive charts implemented via echarts4r, plotly, and JavaScript. Tabular data visualization was achieved using reactable and DT packages. For backend processing, the tidyverse package was used for file reading and standardized data processing, while the parallel package enabled parallel computing and high-concurrency optimization. The DBI package was used for database connection and query. Candidate gene association analyses were performed with the rMVP package (Yin et al. 2021). The ggideogram package was used for the visualization of the genome-wide QTL distribution maps. In the “Tool” module, bcftools and LDBlockShow (Dong et al. 2021) was used for background processing. The LWRR platform was developed on Ubuntu using Docker container technology and deployed on an Elastic Cloud Server to ensure accessibility and scalability.

## Data Availability

The data in this article can be publicly accessed on the website platform at LWRR (https://wheat.dftianyi.com/?page=Download). The genotype data of the 2,191 wheat accessions was available in NGDC under accession number GVM000830. The website interface framework and codes developed by the author are freely open source available for education and academic research at GitHub (https://github.com/CropCoder/LWRR).

## Abbreviations

YR: Yellow Rust
*Pst*: *Puccinia striiformis* Westend f. sp. *tritici*
QTL: Quantitative Trait Locus
GWAS: Genome-Wide Association Study
LWRR: Landscape of Wheat Rust Resistance
BG: Breeding Group
CYR: Chinese Yellow Rust physiological race
π: Nucleotide Diversity
*F*_ST_: Fixation Index
